# Supplementary material for: Antifungal Substances Produced by Xenorhabdus bovienii and Its Inhibition Mechanism against Fusarium solani
Source: Int J Mol Sci. 2022 Aug 12;23(16):9040. doi: 10.3390/ijms23169040 (PMC9409070; doi:10.3390/ijms23169040)
Supplement: Supplementary file 1 [file ijms-23-09040-s001.zip › ijms-1845732-supplementary.pdf]

Supplementary Materials

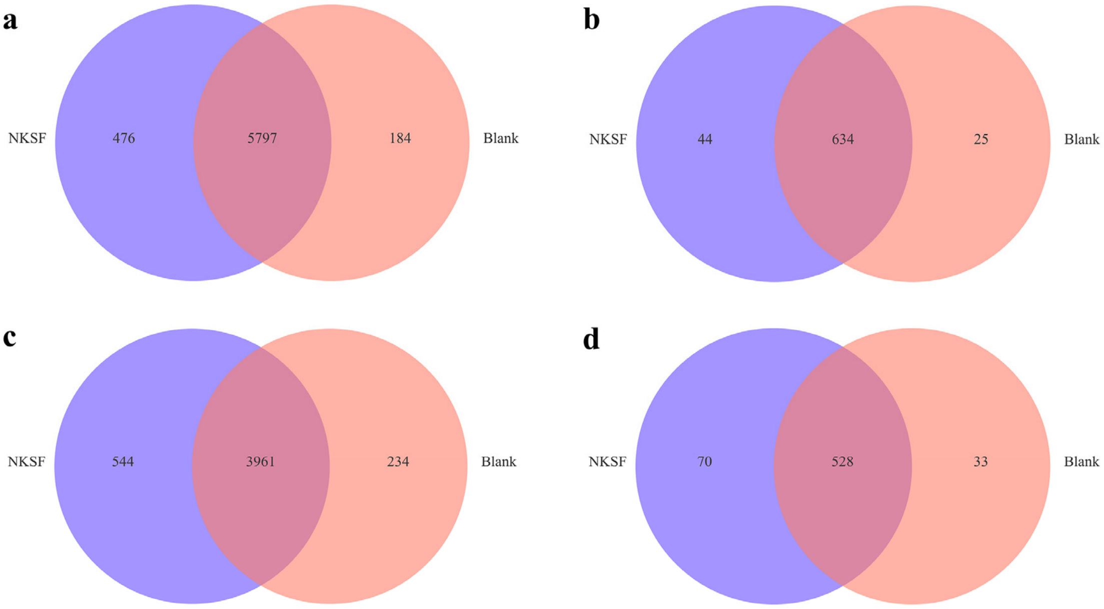

Figure S1. Venn analysis. (a) cationic metabolites; (b) named cationic metabolites; (c) anionic metabolites; (d) named anionic metabolites

Table S1. Number of differential expression genes in KEGG pathway.

KEGG ID	Description	Count	Up	Down
fgr03010	Ribosome	83	82	1
fgr03008	Ribosome biogenesis in eukaryotes	48	43	5
fgr00190	Oxidative phosphorylation	46	36	10
fgr04111	Cell cycle - yeast	41	7	34
fgr04113	Meiosis - yeast	31	3	28
fgr03030	DNA replication	16	0	16
fgr04138	Autophagy - yeast	38	14	24
